# Maxillofacial fracture detection and classification in computed tomography images using convolutional neural network-based models

**DOI:** 10.1038/s41598-023-30640-w

**Published:** 2023-03-01

**Authors:** Kritsasith Warin, Wasit Limprasert, Siriwan Suebnukarn, Teerawat Paipongna, Patcharapon Jantana, Sothana Vicharueang

**Affiliations:** 1 Faculty of Dentistry, Thammasat University, Khlong Luang, Pathum Thani Thailand; 2 College of Interdisciplinary Studies, Thammasat University, Khlong Luang, Pathum Thani Thailand; 3 Sakon Nakhon Hospital, Mueang Sakon Nakhon, Sakon Nakhon Thailand; 4 StoreMesh, Thailand Science Park, Khlong Luang, Pathum Thani Thailand

**Keywords:** Health care, Medical research

## Abstract

The purpose of this study was to evaluate the performance of convolutional neural network-based models for the detection and classification of maxillofacial fractures in computed tomography (CT) maxillofacial bone window images. A total of 3407 CT images, 2407 of which contained maxillofacial fractures, were retrospectively obtained from the regional trauma center from 2016 to 2020. Multiclass image classification models were created by using DenseNet-169 and ResNet-152. Multiclass object detection models were created by using faster R-CNN and YOLOv5. DenseNet-169 and ResNet-152 were trained to classify maxillofacial fractures into frontal, midface, mandibular and no fracture classes. Faster R-CNN and YOLOv5 were trained to automate the placement of bounding boxes to specifically detect fracture lines in each fracture class. The performance of each model was evaluated on an independent test dataset. The overall accuracy of the best multiclass classification model, DenseNet-169, was 0.70. The mean average precision of the best multiclass detection model, faster R-CNN, was 0.78. In conclusion, DenseNet-169 and faster R-CNN have potential for the detection and classification of maxillofacial fractures in CT images.

## Introduction

Maxillofacial injuries are a significant cause of morbidity and are frequently accompanied by serious injuries to other body parts, which represent approximately 533% of major trauma patients^1^. The anatomy of maxillofacial regions is complex with different physical properties including vision, respiration, olfaction, mastication, facial form, etc. Because the components of the maxillofacial skeleton articulate and interdigitate in complex ways, it is difficult to fracture a bone without disrupting its adjacent structures. The pattern of maxillofacial fractures varies depending on the etiology of injury. Common causes of maxillofacial fractures are motorcycle accidents, interpersonal violence, falls from heights, sports, industrial/work related accidents and others/miscellaneous, including gunshot/blast injuries. The mortality rate for maxillofacial trauma is 0.66% and frequently is associated with concomitant injuries including head trauma, which has significant potential for increased morbidity, leads to greater disability and affects the quality of life of the maxillofacial trauma patient^2^. Moreover, the cost of treatment of maxillofacial injuries is relatively high, contributing 28% of the total cost of treating trauma patients, ranging from $793 to $20,678 per patient with hospital stays longer than 2 days^3,4^.

Diagnosis and management of maxillofacial injuries are a challenge, particularly in the context of coexisting multiple traumas in the emergency department. The optimal management of maxillofacial fractures is based on precise clinical and radiographic diagnosis, which sometimes requires immediate intervention and appropriate treatment^5^. Radiologic visualization of the involvement of key anatomic structures is essential to classify the trauma and subsequently apply a differentiated treatment strategy. The objective of treatment is to stabilize and restore three-dimensional facial skeletal anatomy and to provide skeletal support for the proper function of mastication and the function and appearance of the overlying facial soft tissue. Computed tomography (CT) imaging provides detailed information of the specific area for diagnosis and treatment planning by eliminating the superimposition of images that can occur in conventional radiography. The CT image is created using pixels according to its radiosensitivity and is displayed using the Hounsfield scale units, which are compared to known tissue density^6^. In the complex maxillofacial bone region, CT images provide excellent clinical correlation for a suspected abnormality to increase the clinician's ability to accurately diagnose a maxillofacial fracture and eliminate the need for exploratory surgery in maxillofacial trauma cases^7–9^. Combined with advances in computer vision technology, this could help clinicians be faster and more accurate in detecting maxillofacial fractures in CT images for early management, reducing the cost of treatment and preventing morbidity and potential secondary late complications.

Artificial intelligence (AI) involving medical imaging-based deep learning systems has been developed in image feature extraction. Specifically, the convolutional neural network (CNN) has demonstrated enormous potential for extracting clinically important information from medical images. CNN has been successfully applied to detect abnormalities in medical images. Examples include detection of COVID-19^10^, identification of lung nodules^11^, and classification of intracranial hemorrhage via CT images^12^. In traumatic cases, CNN has shown positive results in detecting bone fractures in plain x-rays and CT images with accuracies comparable to or, in some cases, superior to those of experts^13–15^. There are few studies on the adaptation of CNN to detect fractures in maxillofacial regions in CT images, including the detection of mandibular fractures^16^, nasal fractures^17^ and maxillofacial fractures^18,19^. In general, binary classification or detection is used to discriminate or identify whether a lesion is present in the medical image. However, medical abnormalities often appear within different categories in the image. Therefore, multiclass classification or detection could provide specific information or localization of the object of interest in the image^20^. Using a CNN classification model to detect the fracture in the image has the limitation of not being able to determine the exact location of the fracture^18^. Using an object detection model would overcome this limitation and pinpoint the exact location of the fracture in the image^18^. The maxillofacial bone is complex in structure and divided into several regions, such as the frontal, the midface (maxilla, zygoma, naso-orbito-ethmoidal bone) and the mandible. Each region has different facial functions and requires different treatment protocols. The absence of specific localization of maxillofacial fractures could lead to maxillofacial morbidities^5,21^. Therefore, analyzing maxillofacial fractures in CT images through multiclass detection might allow clinicians to specifically locate the site of maxillofacial fractures and enable comprehensive treatment planning of maxillofacial fractures in trauma patients.

The purpose of this study was to evaluate the performance of CNN-based multiclass image classification and detection models for the detection and classification of maxillofacial fractures in CT images. The models are expected to help clinicians with less experience in maxillofacial trauma improve their accuracy, reduce diagnostic errors and time required to detect maxillofacial fractures in CT images for appropriate management of maxillofacial trauma patients.

## Methods

### Ethical approval

This study was approved by the Human Research Ethics Committee of Thammasat University (COA 007/2565) and was performed in accordance with the tenets of the Declaration of Helsinki. Informed consent for the study was waived by the Human Research Ethics Committee of Thammasat University because of the retrospective nature of the fully anonymized images.

### Dataset

This study retrospectively analyzed the CT images data from 150 patients aged 18 years or older in the oral and maxillofacial clinic of regional trauma centers from 2016 to 2020 with a diagnosis of maxillofacial fractures. The inclusion criteria for collecting data from patients aged 18 years or older was due to the data availability in these centers. CT images confirmed maxillofacial fractures were based on a manual review of the clinical and radiological reports recorded in the trauma centers. The maxillofacial CT images were obtained with equipment from different manufacturers using standard imaging protocols. Two-dimensional planar reconstructions were performed in the frontal, sagittal, and oblique planes, parallel to the long axis of the orbits, hard palate and mandible. CT images contained a varied number of axial, sagittal and coronal views with slice thickness of 0.5–2 mm. in increments and a matrix size of 512 × 512 pixels. To develop the maxillofacial fracture detection models, CT images in this study were selected in a two-dimensional axial view of the bone window (window parameters – 2200/200 HU).

A total of 3,407 maxillofacial CT images of 150 patients admitted to trauma centers was divided into CT images containing maxillofacial fractures and CT images without fractures. Of these, 2407 CT images of maxillofacial fracture were distributed to three sites of the maxillofacial area: the frontal fracture of 712 images, the midfacial fracture of 949 images, and the mandibular fracture of 746 images. Another 1000 maxillofacial CT images without fractures were selected from slices of CT images without fracture lines or pathologic lesions.

**Figure 1:**
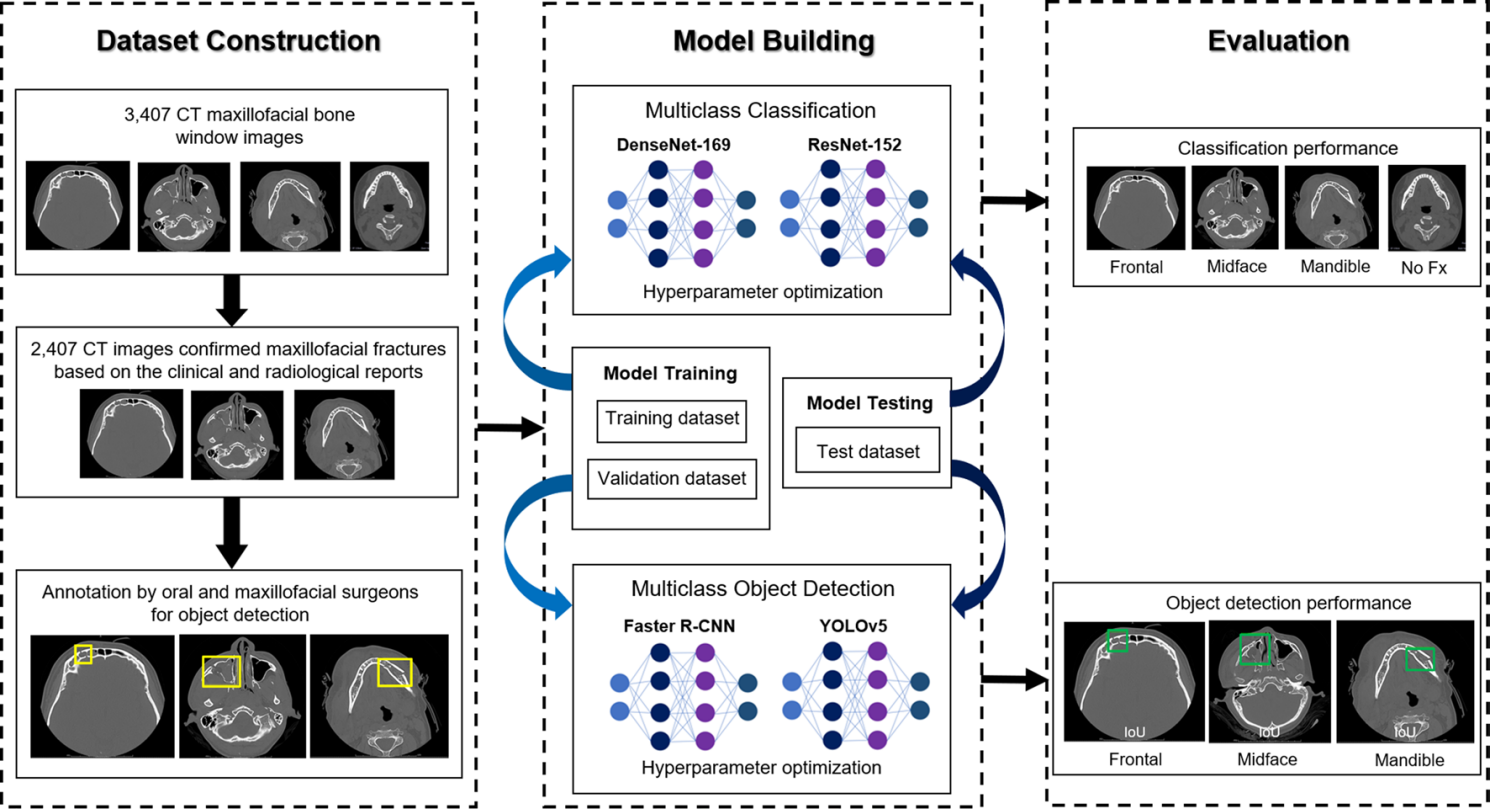
The CNN workflow of data set construction, model building and evaluation. *CNN* convolutional neural network, *CT* computed tomography, *Fx* fracture.

All CT images were uploaded to the VisionMarker^22^ (Digital Storemesh, Bangkok, Thailand) server. VisionMarker is a private web application for image annotation; the public version is available on GitHub (GitHub, Inc., CA, USA). To build the CNN models, the maxillofacial fracture line or ground truth on CT images was reviewed and annotated by consensus of five oral and maxillofacial surgeons with more than 5 years of experience in maxillofacial trauma. For CT images containing maxillofacial fractures, rectangular bounding boxes were drawn around each fracture line and classified as frontal, midface and mandible class according to the location of fracture of frontal, midfacial and mandibular area, respectively (Fig. 1). And for CT images without fracture lines, all images were classified as no fracture. Manual annotations in 3 classes (frontal, midface and mandible) and no fracture (no Fx, without annotation) were used in the learning process of object detection, while multiclass classification (frontal, midface, mandible and no Fx classes) did not require bounding box annotations because the locations were not identified with a classifier. The bounded frontal, midface and mandibular fracture images were split by the accession number into the training, validation, and independent test sets using a 70:10:20 split, with a randomization by distribution to ensure an equal distribution of datasets.

### Experiments

This work was divided into two experiments by deploying well established CNN architectures to create multiclass image classification and object detection models for the classification and detection of maxillofacial fractures in CT images. The workflow of these experiments is illustrated in Fig. 1.

### Multiclass image classification models

The CNN-based classification algorithms, DenseNet-169 and ResNet-152, were adopted to create the multiclass image classification models for identifying the location of maxillofacial fractures as “frontal”, “midface”, “mandible” and “no Fx” for fracture sites that are located at frontal, midfacial, mandibular areas and maxillofacial bone region without fracture line, respectively. Dense Convolutional Network (DenseNet)^23^, a famous image classification algorithm in medical fields, works by embedding each layer with other layers in advance, and can alleviate the vanishing gradient problem, improve feature map propagation, and reduce the number of parameters. Residual Networks (ResNet)^24^ is an architecture implemented by reformulating layers by learning residual functions with reference to layer inputs. This residual learning framework can gain accuracy in classifying objects from the greatly increased depth of the model.

The images were preprocessed by augmentation using Keras ImageDataGenerator (open-source software); then the framework resized an input image to 448 × 448 pixels to feed into a deep learning neural network. DenseNet-169 and ResNet-152 were initialized with ImageNet-pretrained weights with a learning rate of 0.00001 and a batch size of 32. Training was stopped after 12 epochs passed for DenseNet-169 and 15 epochs passed for ResNet-152 without improvement in validation accuracy, and there was no significant indication of over-fitting. All code was implemented in Python utilizing the Tensorflow and Keras packages^14^. Image classification training, validating and testing were done with a Google Colab (Google Inc., CA, USA) using a Tesla P100 (Nvidia Corporation, CA, USA), Nvidia driver: 460.32 (Nvidia Corporation, CA, USA) and CUDA: 11.2 (Nvidia Corporation, CA, USA).

### Object detection models

The object detection algorithms used to construct the maxillofacial fracture detection models were the faster region convolutional neural network (faster R-CNN) and You Only Look Once (YOLO). The faster R-CNN is a two-step detection network that includes a feature extractor, region proposal network (RPN), region of interest (ROI) clustering, and classifier. This algorithm is the combination of the previous object detection system, Fast R-CNN, and RPNs into a single network to share their convolutional features leading to a more real-time object detection method, which improved the speed and accuracy in the object detection compared to basic R-CNN^25,26^. YOLOv5 used in this study is a one-stage object detection algorithm that uses a single network to directly predict object bounding boxes and class probability scores from images. YOLOv5 enables end-to-end training and real time speeds while maintaining high average precision. The detection speed is improved by avoiding the use of RPNs^26–28^.

The images were preprocessed by augmentation using Keras ImageDataGenerator (open-source software); then the framework resized an input image to 256 × 256 pixels for faster R-CNN and 512 × 512 pixels for YOLOv5 to feed into a deep learning neural network. The model was pretrained on ImageNet and COCO (common objects in common) object detection datasets. The training was performed using an on-premise server with 2 of GPU, TitanXP 12 GB (Nvidia Corporation, CA, USA), Nvidia Driver : 450.102 (Nvidia Corporation, CA, USA) and CUDA: 11.0 (Nvidia Corporation, CA, USA) for 20,000 iterations with 0.025 learning rate; 312 epochs; and a batch size of 64 images for faster R-CNN, and 0.01 learning rate; 200 epochs; and a batch size of 8 images for YOLOv5 on the training dataset of CT images with bounding boxes. The training loss was reduced and maintained between 15,000 and 20,000 iterations. Detection accuracy was measured with the intersection over union (IoU) metric between bounding box detection and ground truth and was calculated by a pairwise IoU operation in Detectron^14,29^. For the detection threshold, if the IoU value between the generated bounding box and the ground truth was less than 0.5, then the produced bounding box was considered to be a false detection.

### Model evaluation and statistical analysis

The performance for identification location of maxillofacial fractures in CT images was evaluated by precision, recall, F1 score, sensitivity, specificity and accuracy of the CNN-based multiclass image classification models. The accuracy performance for fracture detection of CNN-based object detection models was evaluated by precision, recall, F1 score, average precision (AP), and mean average precision (mAP). Receiver operating characteristic (ROC) and precision-recall curves were generated using a Python script. An ROC curve plotted by varying the operating threshold was used to assess the ability of the classification model in the discrimination of each class. An ROC curve provided the tradeoff between the sensitivity and 1-specificity. An area under the ROC curve (AUC) was used to summarize the diagnostic accuracy of each class. It was found to have good coverage accuracy over imbalance classes^30^. The statistical analysis for multiclass image classification and object detection was calculated as follows^31^:

$$ {\text{Precision}} = \frac{{{\text{TP}}}}{{{\text{TP }} + {\text{ FP}}}} $$

$$ {\text{Recall }}\left( {{\text{Sensitivity}}} \right) = \frac{{{\text{TP}}}}{{{\text{TP }} + {\text{ FN}}}} $$

$$ {\text{F}}1{\text{ score}} = 2 \times \frac{{\left( {{\text{Precision}} \times {\text{Recall}}} \right)}}{{{\text{Precision}} + {\text{Recall}}}} $$

$$ {\text{Specificity}} = \frac{{{\text{TN}}}}{{{\text{TN }} + {\text{ FP}}}} $$

where the "frontal", "midface" and "mandible" fracture classes are positive classes and the “no Fx” class is a negative class.

- True Positive (TP) is the number of "frontal", "midface", or "mandible" fracture classes that had a correct prediction or detection.
- True Negative (TN) is the number of “no Fx” images that had a correct prediction or detection.
- False Negative (FN) is the number of "frontal", "midface", or "mandible" fracture classes that had no prediction or detection.
- False Positive (FP) is the number of “no Fx” images that had false prediction as fracture class images.

## Results

### Multiclass classification models performance

**Figure 2:**
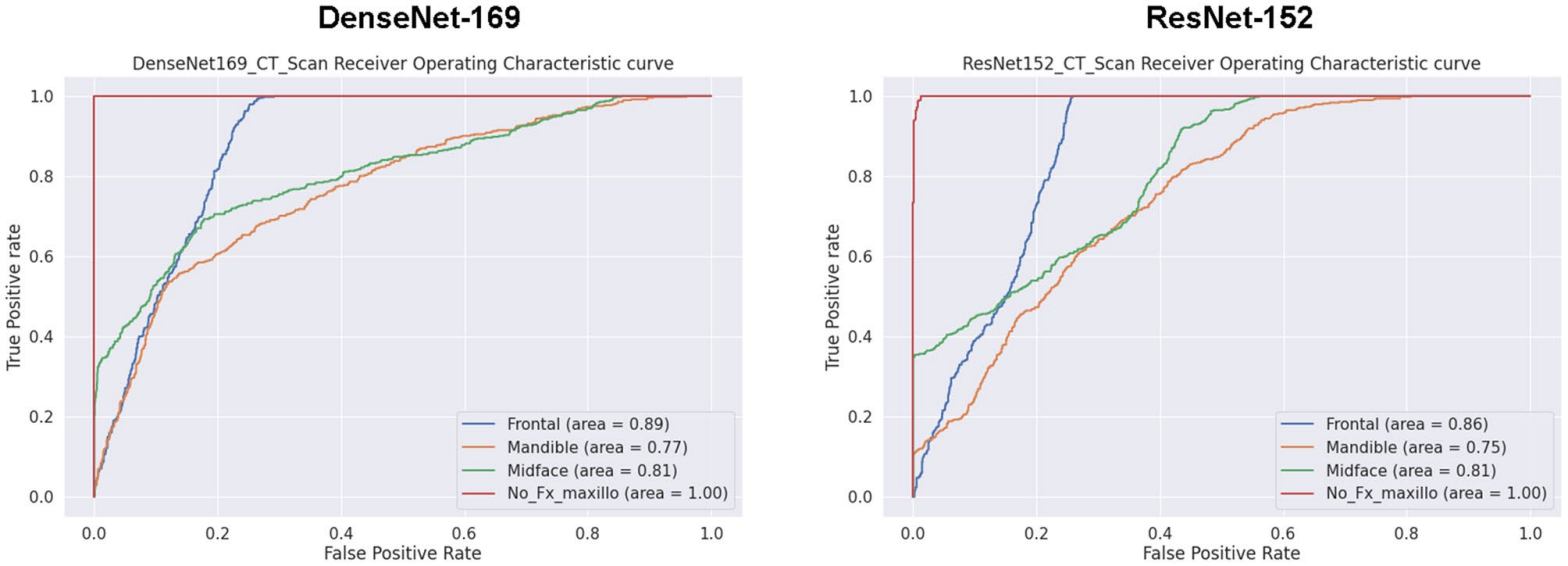
The receiver operating characteristic (ROC) curve for multiclass classification of DenseNet-169 and ResNet-152 in classifying a location of maxillofacial fracture. The AUCs of DenseNet-169 and ResNet-152 were 0.89, 0.86, 0.81; and 0.81, 0.77, 0.75 for frontal, midface, and mandibular fractures, respectively. *AUC* area under the ROC curve, *Fx* fracture.

**Figure 3:**
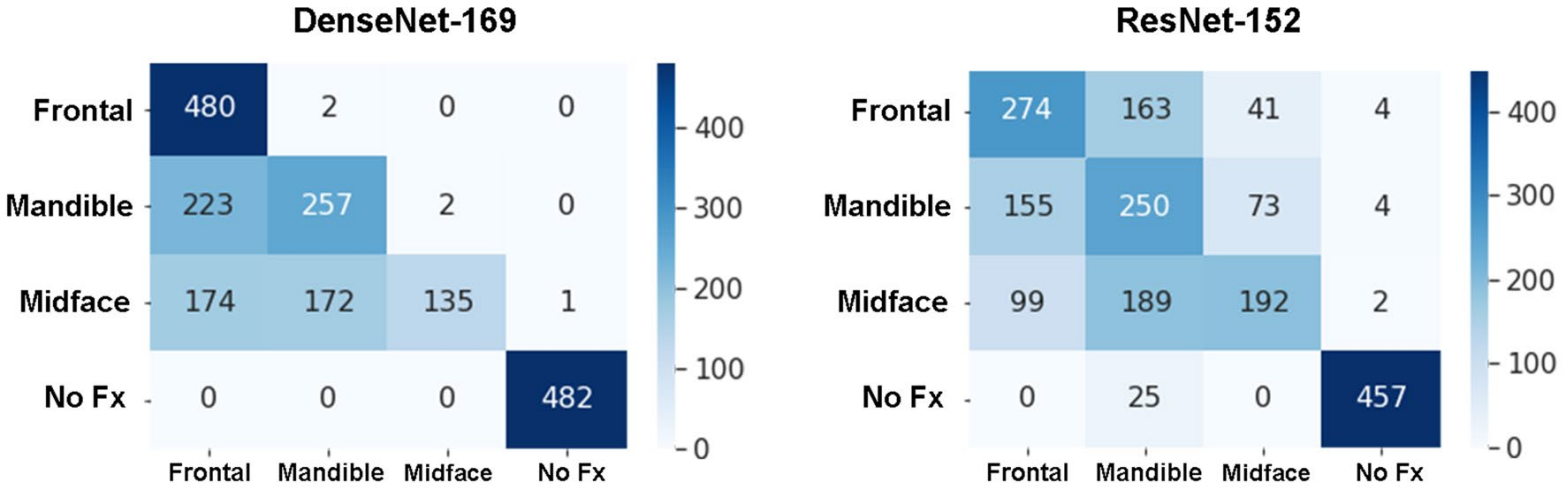
Normalized confusion matrix of multiclass classification of DenseNet-169 and ResNet-152. The y axis represents the true label, and the x axis represents the model’s prediction. *Fx* fracture.

When evaluated on the overall independent test set, the model’s multiclass image classification performance for fracture localization is reported in Table 1. Image classification of DenseNet-169 achieved a precision of 0.55, 0.99, 0.60; a recall (sensitivity) of 1.00, 0.28, 0.53; an F1 score of 0.71, 0.44, 0.56; a specificity of 0.73, 1.00, 0.88; and an accuracy of 0.99, 0.28, 0.53 for classifying frontal, midface, and mandibular fractures, respectively. Image classification of ResNet-152 achieved a precision of 0.52, 0.63, 0.40; a recall (sensitivity) of 0.57, 0.40, 0.52; an F1 score of 0.54, 0.49, 0.45; a specificity of 0.83, 0.92, 0.74; and an accuracy of 0.57, 0.40, 0.52 for classification of frontal, midface, and mandibular fractures, respectively. The overall accuracy of DenseNet-169 and ResNet-152 was 0.70 and 0.61, respectively. For the classification models of imbalance classes, the AUCs of DenseNet-169 and ResNet-152 were 0.89, 0.86, 0.81; and 0.81, 0.77, 0.75 for identification of frontal, midface, and mandibular fractures, respectively. The AUC from 0.75 to 0.89 indicates good agreement with the ground truth. The ROC curves and respective AUC for each fracture site are shown in Fig. 2. A normalized confusion matrix of each multiclass classification model is shown in Fig. 3.

**Table 1:** Multiclass classification performances of DenseNet-169 and ResNet-151 in each class of maxillofacial fractures.

Models	Class	Precision	Recall (Sensitivity)	F1 score	Specificity	AUC of ROC curve	Accuracy
DenseNet-169	Frontal	0.55	1.00	0.71	0.73	0.89	0.99
	Midface	0.99	0.28	0.44	1.00	0.81	0.28
	Mandible	0.60	0.53	0.56	0.88	0.77	0.53
	No Fx	1.00	1.00	1.00	1.00	1.00	1.00
							Overall = 0.70
ResNet-152	Frontal	0.52	0.57	0.54	0.83	0.86	0.57
	Midface	0.63	0.40	0.49	0.92	0.81	0.40
	Mandible	0.40	0.52	0.45	0.74	0.75	0.52
	No Fx	0.98	0.95	0.96	0.99	1.00	0.95
							Overall = 0.61

*AUC* area under the receiver operating characteristic (*ROC*) curve, *Fx* fracture.

### Multiclass object detection models performance

**Figure 4:**
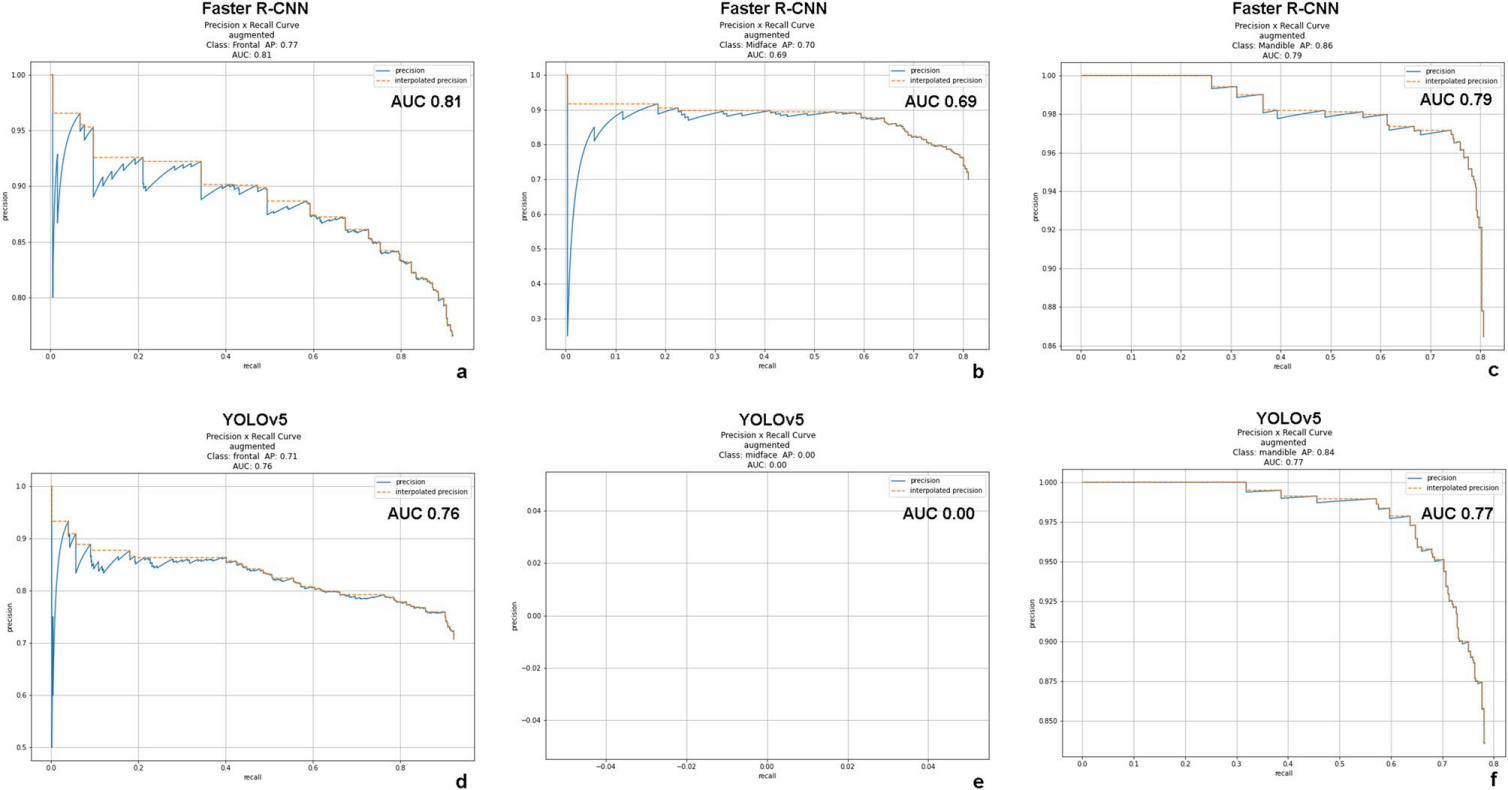
The precision-recall curves for multiclass object detection of faster R-CNN (**a**–**c**) and YOLOv5 (d-f) for detecting a location of maxillofacial fracture. The AUC of the faster R-CNN and YOLOv5 were 0.81, 0.76, 0.69 and 0.00, 0.79, 0.77 for detection of frontal (**a**, **d**), midface (**b**, **e**), and mandibular (**c**, **f**) fractures, respectively. *AUC* area under the precision-recall curve.

**Figure 5:**
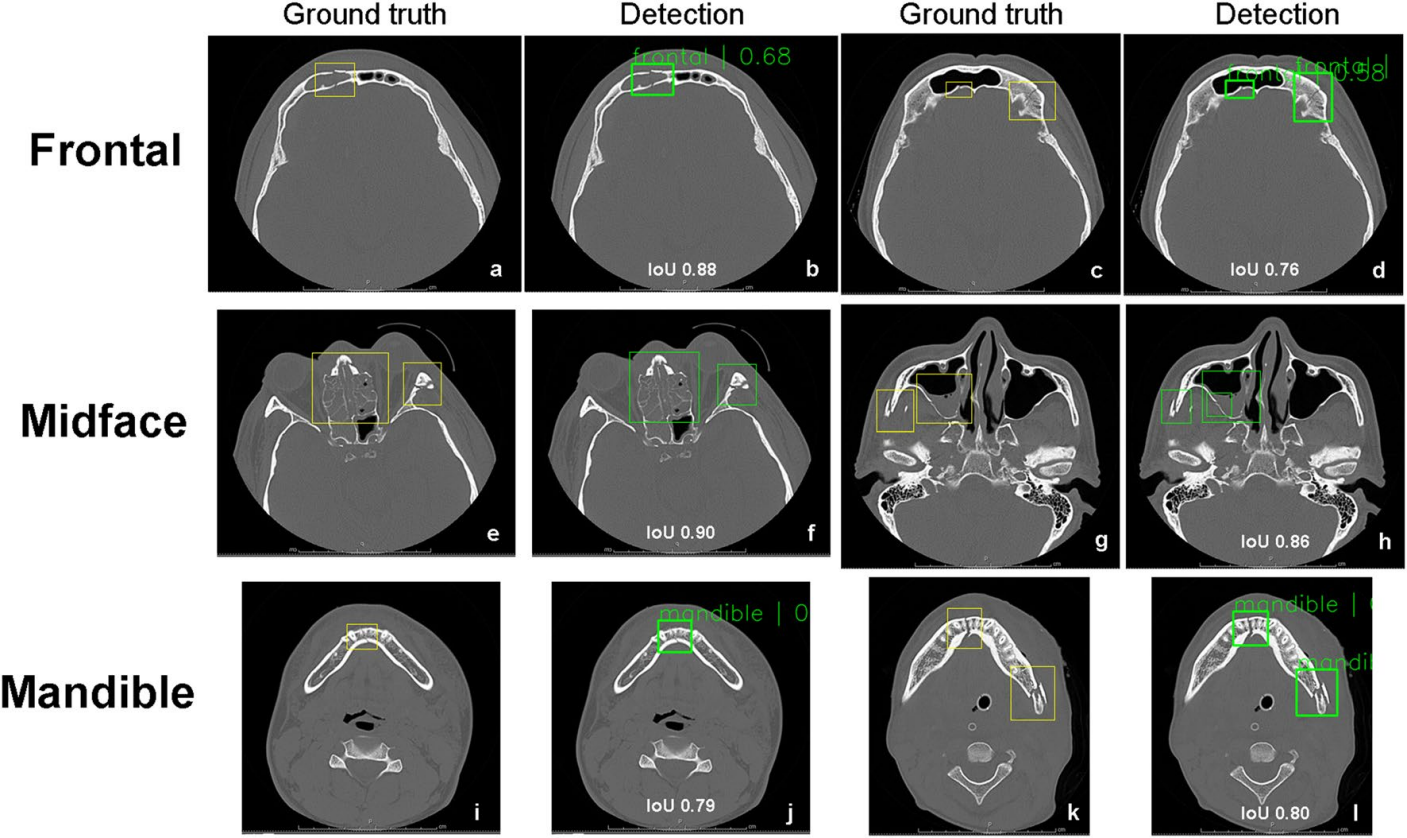
Examples of ground truth and detection bounding boxes of faster R-CNN on CT images from an independence test set. The yellow boxes represent boxes annotated manually by oral and maxillofacial surgeons of frontal (**a**, **c**), midface (**e**, **g**), and mandibular (**i**, **k**) fractures. The green boxes are the true positive output for detection of frontal fractures with IoU of 0.88 and 0.76 (**b**, **d**), midface fractures with IoU of 0.90 and 0.86 (**f**, **h**), and mandibular fractures with IoU of 0.79 and 0.80 (**j**, **l**). *IoU* Intersection over union.

The trained object detection algorithms correctly identified each maxillofacial fracture labeled in the independent test dataset with high accuracy on the automatically generated bounding boxes. The results are shown in detail in Table 2. Detection performance of the faster R-CNN achieved a precision of 0.77, 0.70, 0.86; a recall of 0.92, 0.81, 0.81; an F1 score of 0.84, 0.75, 0.83; and an AP of 0.82, 0.72, 0.79 in detection of maxillofacial fracture locations at frontal, midfacial, and mandibular area, respectively. Detection performance of the YOLOv5 achieved a precision of 0.71, 0.00, 0.84; a recall of 0.92, 0.00, 0.78; an F1 score of 0.80, 0.00, 0.81; and an AP of 0.77, 0.00, 0.77 in detection of maxillofacial fracture locations at frontal, midface, and mandibular area, respectively. The mAPs of faster R-CNN and YOLOv5 models for detecting the maxillofacial fractures were 0.78 and 0.51, respectively. The AUCs of the faster R-CNN and YOLOv5 were 0.81, 0.76, 0.69 and 0.00, 0.79, 0.77 for detection of frontal, midface, and mandibular fractures, respectively (Fig. 4). Examples of faster R-CNN detection outputs on maxillofacial CT images are shown in Fig. 5.

**Table 2:** Multiclass object detection performances of faster R-CNN and YOLOv5 for identification of maxillofacial fracture in each class.

Models	Class	IoU (threshold)	Precision	Recall	F1 score	AUC of precision-recall curve	AP
Faster-RCNN	Frontal	0.5	0.77	0.92	0.84	0.81	0.82
	Midface	0.5	0.70	0.81	0.75	0.69	0.72
	Mandible	0.5	0.86	0.81	0.83	0.79	0.79
							mAP = 0.78
YOLOv5	Frontal	0.5	0.71	0.92	0.80	0.76	0.77
	Midface	0.5	0.00	0.00	0.00	0.00	0.00
	Mandible	0.5	0.84	0.78	0.81	0.77	0.77
							mAP = 0.51

*IoU* intersection over union, *AUC* area under the precision-recall curve, *AP* average precision, *mAP* mean average precision.

## Discussion

This study demonstrated the high performance of multiclass CNN models in detecting the maxillofacial fracture locations in CT maxillofacial bone window images. To our knowledge, this represents the first report of multiclass classification and detection of maxillofacial fractures by deep learning in the literature. The promising results achieved are notable given the limited size of the training data set, which was only 3,407 CT images. This study selected only one view of the CT image to develop the CNN model, which was an axial view of the maxillofacial bone window CT image. In normal clinical situations, CT images of maxillofacial bone need to be reviewed at all views to complete the diagnosis of maxillofacial fractures. For the most reliable ground truth, all locations of maxillofacial fractures on CT images, which have been labeled by oral and maxillofacial surgeons, have been confirmed by clinical and radiological reports. The maxillofacial bone is a complex structure and related to several functions, including vision, olfaction, respiration, mastication, etc. Therefore, missing a maxillofacial fracture diagnosis could become a potential morbidity^2,5^. Additionally, locating the maxillofacial fracture site automatically via multiclass CNN models would be beneficial for maxillofacial trauma surgeons to have more traumatic information, reduce misdiagnosis and select the most appropriate treatment for the maxillofacial trauma patient.

Previous studies using CNN-based classification models to identify maxillofacial fractures in CT images^16,17,19^ have reported the performance of binary classification with the AUC ranging from 0.82 to 0.96. To further investigate the potential of CNN-based classification models as a multiclass classification technique, this study found that DenseNet-169 yielded high classification performance in classifying the maxillofacial fractures at frontal area with an AUC and accuracy of 0.89 and 0.99, respectively. Unfortunately, both DenseNet-169 and ResNet-152 achieved unsatisfactory performance in classifying maxillofacial fractures in CT images with an AUC and accuracy of 0.28, 0.53 and 0.40, 0.52 for midfacial and mandibular area, respectively. The characteristic of fracture lines, which are relatively small and located in larger structures in the midface and mandibular areas, could be the main reason for the difficulties in fracture classification.

For CNN-based objection detection model, the results of this study achieved high accuracy in detecting the location of maxillofacial fracture in CT images with an mAP of 0.78 for faster R-CNN but relatively low performance for YOLOv5 with an mAP of 0.51. The results were consistent with previous studies showing that the accuracy of the one-stage algorithm for detecting small targets was not as good as the two-stage algorithm^27,28^. A recent study trained data on nasal fractures using YoLoX-S to detect maxillofacial fractures in various regions in CT images^18^. Binary detection performance yielded an AP ranging from 0.5 to 0.7 by varying data augmentation methods. In the study, YOLOv5 was trained using data from various maxillofacial fracture regions, including frontal, midface and mandibular fractures, to build the multiclass detection model. The model was able to detect frontal and mandibular fractures with an AP of 0.77. However, YOLOv5 could not detect midface fractures (an AP of 0.00), which are typically small compared to the complex and large structure of the midface in CT images.

Deep CNN's high performance in detecting maxillofacial fractures may in part stem from the fact that the machine does not suffer from concentration decline, and is consistent and reproducible when presented with the same input data, unlike humans, who are susceptible to error in detecting maxillofacial fracture in CT images due to limited concentration^32^. The CNN model can potentially be trained with a vast amount of training data, far more than any maxillofacial trauma surgeon will experience in their lifetime, resulting in an unparalleled possibility of CNN. This automated maxillofacial fracture location identification system could be implemented in different ways depending on the needs of the individual clinical environment, which could act as a new form of “preliminary” reporting and automatically alert clinicians concerned with the presence and location of maxillofacial fractures. This can be especially useful in hospitals that currently lack coverage for maxillofacial trauma surgeons or radiologists. As this automated process could be upgraded and executed almost instantaneously in real time, it can save considerable time by avoiding diagnostic errors and delays in preliminary interpretation. This system can improve not only emergency department efficiency, but also patient outcomes by reducing detection errors and time to treatment.

There are limitations to this study. First, the CT image data for the experiments was retrospective data from only two institutions, potentially limiting its generalizability, and the CT image resolution was not high quality with 512 × 512 pixels, which may be the cause that limits the development of accurate maxillofacial fractures classification models. For future work, collecting CT image data from multi-institutional centers with many different types of CT machines and higher resolution image data would improve the diagnostic performance of the model, which should allow generalizing the use to multi-trauma centers. Additionally, other views of CT images, including coronal and sagittal views, should include an upgrade of the CNN model to be better in analyzing and localizing the site of maxillofacial fractures. Furthermore, to be more specific to the localized site of maxillofacial fractures, multiclass analysis of the midfacial area, which is complex and composed of multi-bony structures, could be another step to further develop this CNN model to be applied in the real clinical setting.

In conclusion, this study demonstrated that the faster R-CNN, a multiclass object detection CNN-based model, has great potential to specifically detect the maxillofacial fracture locations in CT images. However, the small line in the large structure of maxillofacial fractures resulted in moderate to low accuracy of CNN-based models of multiclass classification in classifying each location of maxillofacial fractures, except DenseNet-169 which achieved high accuracy in the classification of fracture lines at the frontal area in CT maxillofacial bone images. The models are expected to be the AI-based diagnostic aid tool in identifying maxillofacial fractures in CT images to increase accuracy, provide additional information, and reduce diagnostic time and errors for clinicians managing patients with maxillofacial trauma.

## Data Availability

The datasets used and analyzed during the current study are available from the corresponding authors on reasonable request.
